# Public health prevention and emergency preparedness funding in the United States: Are we ready for the next pandemic?

**DOI:** 10.1016/j.amsu.2020.10.007

**Published:** 2020-10-10

**Authors:** Brendon Sen-Crowe, Mark McKenney, Adel Elkbuli

**Affiliations:** Department of Surgery, Division of Trauma and Surgical Critical Care, Kendall Regional Medical Center, Miami, FL, USA; Department of Surgery, Division of Trauma and Surgical Critical Care, Kendall Regional Medical Center, Miami, FL, USA; Department of Surgery, University of South Florida, Tampa, FL, USA; Department of Surgery, Division of Trauma and Surgical Critical Care, Kendall Regional Medical Center, Miami, FL, USA

**Keywords:** Emergency preparedness, Public health policy, Pandemics, Center for disease, Control and prevention, Public health funding

## Abstract

• The CDC's cumulative funding for Public Health, Prevention, and Emergency Preparedness decreased over the course of 2011–2020, however, NIH funding dedicated to Prevention displayed an overall increase from 2008 to 2019.
• The Hospital Preparedness Program (HPP) is the only source of federal funding for healthcare system readiness, yet their budget exhibited consistent reductions from 2003 to 2018.
• Public health emergencies like the COVID-19 pandemic have demonstrated more significant consequences than other diseases that receive greater funding.
• Allocating additional funding towards CDC health prevention in addition to expanding the Public Health Preparedness Response Fund (PHPR) and Prevention and Public Health Fund (PPHF) may improve future prevention and preparedness measures.

Public health threats of are escalating. The US spends <3% of healthcare spending towards public health and prevention [[1], [2], [3]]. Most of the funds transit through the 10.13039/100000030CDC and smaller amounts through Health and Human Services (HHS) and 10.13039/501100003526Department of Agriculture (10.13039/100000199USDA). In addition, the Prevention and Public Health Fund (PPHF) supports prevention and public health programs [1]. The 10.13039/100000002NIH provides research funding for public health prevention [2]. Finally, the Hospital Preparedness Program (HPP) is the only source of federal funding for healthcare system readiness [3].

Public health funding is deficient when it is required most. COVID-19 demonstrates this, as it continues to climb closer to the leading cause of US daily deaths [4]. The CDC's annual operating plans from 2010 to 2020 were reviewed along with the NIH's estimates of funding from 2008 to 2020 and HPP's Funding from 2003 to 2018 [2,3]. The amount of funding allocated to public health and their disciplines was analyzed and compared between the three organizations.

The CDC's cumulative funding has been decreased over the last decade (Fig. 1a). In 2010, the cumulative public health budget was $10.88 billion and decreased to $7.97 billion as of 2020 (Fig. 1a). The 10.13039/100000030CDC has 7 main categories that fall under Public Health, Prevention and Emergency Preparedness: 1) Preventative Health and Health Services Block Grant (PHHSBG), 2) Public Health Scientific Services (PHSS), 3) Global Disease Detection and Emergency Response (GDDER), 4) Public Health Leadership and Support (PHLS), 5) Public Health and Social Services Emergency Fund (PHSSEF), the 6) Prevention and Public Health Fund (PPHF) and the 7) Public Health Preparedness Response Fund (10.13039/100005195PHPR) (Fig. 1b).

**Fig. 1a:**
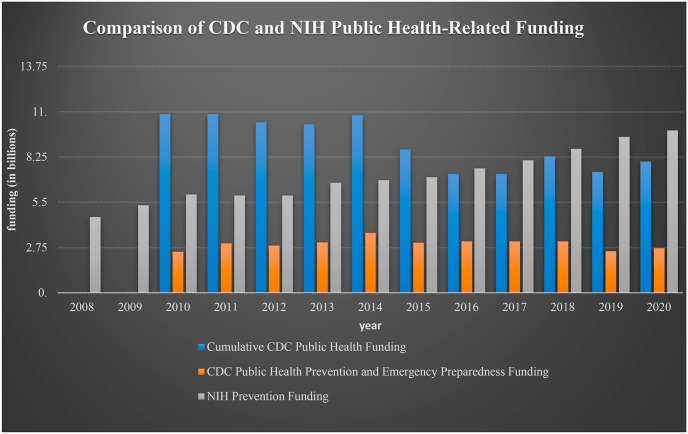
**The CDC funding allocated towards Public Health, Prevention, and Emergency Preparedness decreased from 2011**–**2020**. The cumulative public health funding decreased from $10.87 billion in 2010 to 7.34 billion by 2019, followed by an increase to $7.97 billion in 2020. In contrast, the NIH Prevention funding steadily increased from 5 billion in 2008 to $9 billion by 2019. The estimated NIH Prevention funding for 2020 is $10 billion.

**Fig. 1b:**
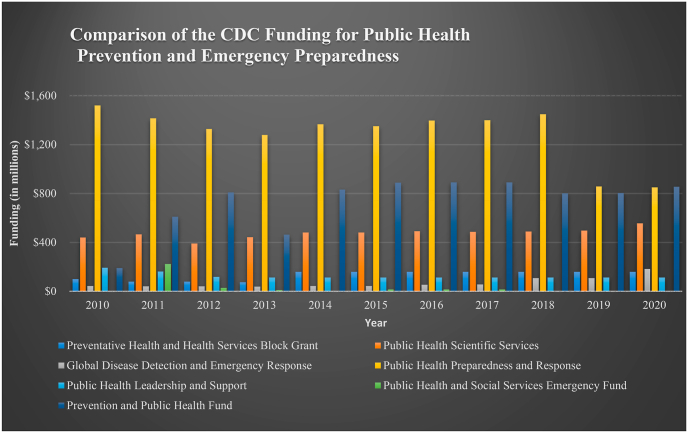
**Categories contributing to the overall CDC Public Health Prevention and Emergency Preparedness vary widely.** The Public Health Preparedness and Response funding decreased from $1522 million in 2010 to $850 in 2020. Similarly, the Public Health Leadership and Support revealed reduced funding from $194 million in 2010 to $114 million by 2020. In contrast, Preventative Health and Health Services Block Grant, Public Health Scientific Services, Global Disease Detection and Emergency Response, Public Health and Social Services Emergency Fund, and the Prevention and Public Health Fund steadily increase from 2010 to 2020.

The funding sums varied by year and category but when evaluated in total there was an overall decrease in the funding dedicated towards Public Health Prevention, and Emergency Preparedness from $3.00 billion in 2011 to $2.73 billion in 2020 [1].

NIH public health prevention funds was approximated using the budget for the Prevention category. The funding consistently increased from $4.6 billion in 2008 to $9.5 billion in 2019 (Fig. 1a). The estimated budget for 2020 is $9.9 billion and a projected budget decrease in 2021 ($9.1 billion) [2]. The budget dedicated towards healthcare preparedness shows a consistent decrease in funding (Fig. 2), from $498 million in 2003 to ~$227 million by 2018 [3].

**Fig. 2:**
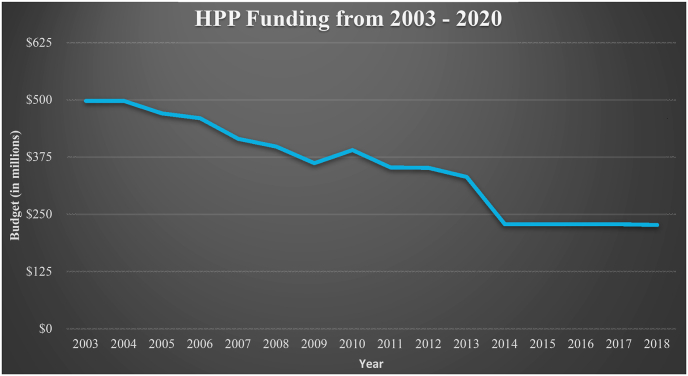
**HPP Demonstrates an Overall Decrease in Funding.** The funding recorded a high of $498 million in 2003 and steadily decreased to $362 million in 2009. Following a brief increase in funding in 2010 ($391 million), the funding consistently decreased to $227 million by 2018.

The CDC's Public Health Emergency Preparedness (PHEP) Cooperative funding, a subcategory of the 10.13039/100005195PHPR, was reduced 30% over 18 years [1,2]. PHEP is the primary source of federal funding for state public health and emergency response.

HPP funding has consistently demonstrated reductions from 2003 to 2018 (Fig. 2). Similarly, the funding for the CDC's 10.13039/100005195PHPR has decreased since 2011 (Fig. 1b, Fig. 3).

**Fig. 3:**
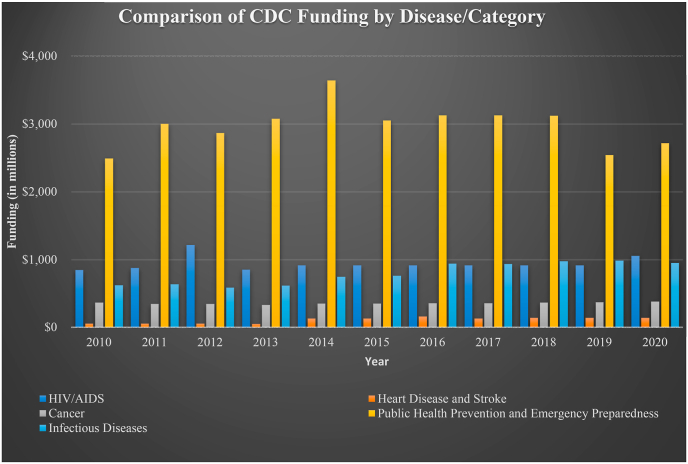
Public Health Prevention and Emergency Preparedness Funding Decreases after 2014. The funding for public health prevention and emergency preparedness initially increased from $2494 million to $3644 in 2014, however, decreased to a low of $2541 million by 2019. In contrast, the HIV/AIDS, Cancer, Infectious Disease and Heart Disease and Stroke funding increased overall between 2010 and 2020.

The impact of public health emergencies cannot be overlooked. As of September 10, 2020, there were 191,766 deaths due to COVID-19 in the US, over 12 times the amount of annual deaths due to HIV/AIDS in two-thirds of the time [4]. Yet, the funding for HIV/AIDS continues to increase, while funding for the CDC's public health prevention continues to decrease (Fig. 3).

There have been many public health emergencies in the past to support an increase in public health funding. The high fatality burden COVID-19 places on the US should be recognized, and the funding matched to the damage inflicted on the population and healthcare system. Ebola infected over 27,000 people worldwide, leading to 11,000 worldwide deaths, prompting the implementation of a $1.76 billion funding budget dedicated to Ebola Response and Preparedness [1]. Moreover, other public health emergencies stress the importance of public health prevention and emergency preparedness such as the Zika outbreak. From 2015-present, Zika virus has infected 43,194 people in the US and is transmitted by mosquitos [1].

A couple recommendations can be made. Improve emergency preparedness by increasing the CDC's funding for emergency preparedness programs. Increasing the PHEP funding will give core resources to local and state entities. Next, increase HPP funding. Since 2003, HPP funding has been reduced by more than 50% (Fig. 2), creating significant obstacles in their ability to support the healthcare system during periods of emergency. Finally, the PPHF funding can be expanded so that the vaccine infrastructure and surveillance capacity are adequately supported. In the case of the PPHF, prevention should not be sacrificed for treatment funding. One feasible strategy to limit a public health crisis is to prevent and inhibit the course of the crisis from the beginning, rather than merely reacting to the burden placed on the nation. If funding to the agencies that foster prevention and preparedness measures can be increased, we may have an opportunity to prevent the next public health emergency.

Public health emergencies place a significant burden on the nation and yet, the CDC Public Health and HPP funding continues to decrease. Increasing the HPP, CDC Public Health funding, and other public health bills provides an avenue to pursue in order to lessen the burden public health emergencies such as the COVID-19 pandemic place on the nation.

## Ethical approval

Not applicable.

## Sources of funding

None.

## Author contribution

Study design and conception: Adel Elkbuli.

Data collection, interpretation and analysis: Brendon Sen-Crowe, Adel Elkbuli, Mark McKenney.

Manuscript preparation: Brendon Sen-Crowe, Adel Elkbuli.

Critical revision of manuscript: Brendon Sen-Crowe, Adel Elkbuli, Mark McKenney.

All authors read and approved the final manuscript.

## Research registration Unique Identifying number (UIN)

Name of the registry:

Unique Identifying number or registration ID:

Hyperlink to the registration (must be publicly accessible):

Not applicable-no human subjects or research participants’ data were utilized or collected.

## Guarantor

Adel Elkbuli.

Mark McKenney.

## Provenance and peer review

Not commissioned, editor reviewed.

## Declaration of competing interest

Authors declare no competing interests.

## References

[1] Prevention and Public Health Fund. Centers for disease control and prevention. https://www.cdc.gov. Accessed September 10, 2020.

[2] NIH Categorial Spending – Estimates of Funding for Various Research, Condition, and Disease Categories. U.S. Department of Health & Human Services. https://report.nih.gov/categorical_spending.aspx. Accessed September 10, 2020.

[3] Hospital Preparedness Program. U.S. Department of Health & Human Services. https://www.phe.gov/Preparedness/planning/hpp/Documents/hpp-intro-508.pdf. Accessed September 10, 2020.

[4] (January 22, 2020). COVID-19 Map.

